# Validity and Internal Consistency of the Preschool-FLAT, a New Tool for the Assessment of Food Literacy in Young Children from the Training-To-Health Project

**DOI:** 10.3390/ijerph17082759

**Published:** 2020-04-16

**Authors:** Garden Tabacchi, Giuseppe Battaglia, Giuseppe Messina, Antonio Paoli, Antonio Palma, Marianna Bellafiore

**Affiliations:** 1 Department of Psychology, Educational Science and Human Movement, University of Palermo, 90144 Palermo, Italy; giuseppe.battaglia@unipa.it (G.B.); giuseppe.messina17@unipa.it (G.M.); antonio.palma@unipa.it (A.P.); marianna.bellafiore@unipa.it (M.B.); 2 Department of Biomedical Sciences, University of Padova, 35121 Padova, Italy; antonio.paoli@unipd.it

**Keywords:** food literacy, validity, consistency, preschool education, assessment, structural equation modeling

## Abstract

Background: The importance of assessing “food literacy” since youth has been highlighted and, to this purpose, valid and consistent instruments are needed. This study aimed to assess the validity and internal consistency of the preschool-FLAT (Food Literacy Assessment Tool). Methods. 505 children from 21 kindergartens, recruited within the Training-to-Health Project in Palermo (Italy), underwent oral sessions and activities on food-related aspects. Their knowledge/skills were recorded in the preschool-FLAT. The following scale measures were assessed: Content validity; internal consistency (Chronbach’s alpha coefficients); construct validity (Structural Equation Modeling—SEM); discriminant validity (intervention subgroup of 100 children vs. control group of 27 children). Results. Acceptable content validity of a 16-items scale and overall adequate internal consistency were revealed: Content validity index (CVI) 0.94, content validity ratio (CVR) 0.88, Chronbach’s alpha 0.76. The SEM revealed a 4-factor model fitting the data well (comparative fit index 0.939, root mean square error of approximation 0.033). Discriminant validity was good (intervention group scoring higher than control, *p* < 0.001, unpaired Student’s t-test). Conclusion. The preschool-FLAT revealed good psychometric properties, adequate validity and internal consistency. This is the only instrument in the literature specifically targeted to 3–6 years old children that could be effectively used to assess food literacy.

## 1. Introduction

Previous studies have demonstrated that children in preschool age are able to comprehend concepts of food, nutrients and energy, to recognize healthy and non-healthy foods, and can accumulate and process information by observing familiar adults and teachers; thus, they can take advantage from nutrition education within a preschool context by improving their knowledge and skills related to nutrition [1,2].

Children in distinct cognitive stages think, decide and perceive food topics differently. Children in preschool age are in a pre-operational stage; e.g., they do not make a distinction between foods and snacks, they think that the ingested food goes into the stomach and do not change in the body, or they could mention foods that are healthy, but they could not explain why [1].

Early childhood intervention programs including stimulation component are generally more effective on cognition than nutritional interventions alone. Children are more attracted by stimulus material such as visible and physical characteristics like texture, shape, or color to describe food, pictorial prompts, tasting and cooking sessions, as they are interactive ways to engage children directly and can help researchers control for limited verbal abilities and timidity [3]. Moreover, pre-schoolers can better learn by experimentation, rather than by passive listening, and structured play-based activities (such as meal planning, food preparation, and serving food) can be an excellent way of learning and of assessing them for their food knowledge and skills [4,5,6,7,8].

Therefore, it is possible to help children in developing their food-related literacy that is important in shaping their future correct food choices and preventing nutrition-related diseases, which determine high morbidity and mortality in the population [9]. “Food literacy” (FL) is a current term used for indicating knowledge, skills, and behaviors related to food and nutrition [10,11,12]. A recent review reports that food decisions reflect the application of knowledge, information and skills to make food choices [13]. Furthermore, in recent years, an increasing number of studies underlies how FL could impact on body image since preschool age; e.g., having a well-developed food literacy could encourage attribution of negative characteristics to fat shapes and then help to make choices that allow attainment of a correct weight status. This has a consequence on the development of social competences including social values and social desirability [14,15]. In detail, this effect seems to be mediated by a wide range of individual and sociocultural factors encompassing body satisfaction vs. dissatisfaction, body mass index (BMI), a positive thin ideal internalization vs. attributing negative characteristics to fat shapes, a higher maternal dietary restraint, peers’ influence and media exposure [16].

The importance of assessing FL since preschool age has been highlighted and, to this purpose, reliable and valid measures are needed. Existing FL measurement tools tend to emphasize literacy and numeracy skills and/or nutrition knowledge [12]; some of them employ only nutrition knowledge-based outcomes [17,18]; or they are targeted to adults or scholar children [19,20,21,22,23]. Hence, it is urgent to develop a tool that is targeted to pre-schoolers, that is accurate and reliable and assesses not only knowledge but also skills related to food and nutrition. Determining the validity and consistency of a psychometric instrument that measures a construct such as FL, and that helps in evaluating childhood obesity prevention interventions, is therefore necessary [24,25,26,27,28].

In this context falls the *Training-to-Health* Project [29], which, among the general purpose of educating pre-schoolers to a healthy lifestyle, aimed at developing a tool for the assessment of FL in pre-schoolers to be inserted in a particular curriculum program delivery in the food and nutrition context. The present study reports on the content, construct and discriminant validity of this tool, and on its internal consistency.

## 2. Materials and Methods

### 2.1. Participants

For the present study a sample of 505 children aged 3–6 years was selected. These pre-schoolers were recruited from twenty-one kindergartens placed in areas with different socio-economic conditions, under the Palermo City Council administrative boundaries. The socio-economic environment (SEE) of the schools was evaluated through the “index of socio-economic disadvantage”, measured on the basis of four indicators of deprivation in the 55 different city districts. These indicators were the unemployment rate, employment rate, youth concentration rate, and schooling rate. The SEE was then divided in two classes (low/medium and medium/high) for the sake of this study.

### 2.2. Preschool-FLAT

The preschool-FLAT (Food Literacy Assessment Tool) is a tool-kit for the assessment of FL in pre-schoolers, and was created by the Training-to-Health team [30], according to the educational objectives of the Italian Ministry of Education, University and Research (MIUR) targeted to kindergartens and primary schools (e.g., body and movement; images, sounds, colors; speeches and words; world’s knowledge; the self and the other) and also on the basis of the FL knowledge and skills components described by Vidgen et al. [11].

Initially, a panel of five experts in the field of nutrition, education, psychology, and public health proposed a total of 20 items regarding plausible abilities that children of preschool age can develop, according to personal expertise in the field and to previous literature describing the knowledge and skills potentially reachable in this age. In this view, the model form Vidgen et al. [11] was slightly modified by the authors, to adapt the knowledge and skills that children in this age can likely acquire. These 20 items were settled and gathered in 5 domains, each including 4 items, having a specific objective and together reflecting constructs of food knowledge and skills.

Domain 1 aimed at assessing the relationship between weight status and food, and between weight status and health. Children were advised on the meaning of overweight and obesity, and of good health status; they were tested on the ability of discriminating between different weight status images, relating overweight and obesity to the concept of health, and associating food to incorrect weight status.

Domain 2 assessed the relationship between food quality/quantity and health, with different activities aimed at learning the main food categories; knowing, recognizing and naming the healthy and non-healthy foods; being able to weight foods; discriminating different quantities (small, medium, big portions); learning the relationship between portion and health; knowing color of foods and relationships between food color and health.

Acquiring the relationship between food and environment was the aim of Domain 3 that had different activities: Learning the meaning of organic food; recognizing the packed organic products; identifying food seasonality. The organic food in this context has a particular importance, since Sicily is the main producer of organic foods in Italy, and an increased sale rate sale in the large distribution has been recorded in the last years.

Domain 4 regarded traditional foods; children were made aware of the Mediterranean diet, the typical Sicilian food; they performed activities to recognize smells and flavors of typical foods; they experimented to knead water and flour to create shapes of typical foods; they became able to assemble a meal by choosing typical foods. A total of 4 items were included.

The last Domain 5 had the main objective of making children aware of the distribution of foods in the different daily meals, able to build the food pyramid and associate foods in the different pyramid levels.

A copy of the proposed tool is placed in the Supplementary Material.

### 2.3. Procedure

Preschool-FLAT was used for a total of ten hours during four months from January to May 2016. A team composed of physical education experts performed within the schools, with the support of the class teacher, a total of five laboratory sessions, each session corresponding to a domain of the preschool-FLAT. The team was initially trained for ten hours in order to standardize the methodologies and the process of assessment, including weight and height measurements and FLAT assessment. A previously prepared study protocol was showed during the training sessions, reporting general and specific objectives, the activities to perform and the evaluation sheet to be administered to participating children for each domain. The Ethical Board of the Azienda Ospedaliera Universitaria Policlinico Paolo Giaccone Palermo (Palermo 1, N. 02/2018) approved the study. The criteria stated in the Declaration of Helsinki were followed. Parents were invited to sign consent forms to allow their children participating in the study.

The preschool-FLAT tool was used in the laboratory sessions organized in the classes. As indicated in the tool, each session of the food laboratory consisted of a brief oral session (around twenty minutes), using story-telling methodology, aimed at providing children with information on the topic of the domain; subsequently, practical sessions were conducted, where children were invited to perform activities related to the previously explained arguments. The operators assessed children’s abilities to perform the tasks by using the “evaluation part” of the sheets during the activities or at the end of them; e.g., if the child had to paste the correct portion close to the right emoticon (a smiley or a sad face), he did it in the sheet that was pre-arranged with the necessary material (Supplementary Material).

After the administration, scores were calculated in each evaluation sheet for the different activities. For each domain, a 5-point scale (from 0 to 4) according to Likert was possible. A total score ranging from 0 to 20 (where 0 indicates no FL and 20 indicates high FL) and obtained by the sum of the scores of the single items was generated in order to rank children in classes of FL.

Operators transferred data from the hardcopies to excel files in order to perform the statistical analyses.

### 2.4. Data Analysis

#### 2.4.1. Sample Characteristics

Distribution of the data was assessed though the Skewness/Kurtosis test for Normality. The characteristics of the sample were measured in number and percentages for categorical variables, and means and SD for normally distributed continuous data.

Since the FL score was normally distributed, crude scores were standardized into z-scores in order to identify children ranking below and over 1 SD and classify them into low FL level and high FL level, respectively. All data were analyzed by using STATA/MP 12.1 (StataCorp).

#### 2.4.2. Content Validity

In order to catch the most important aspects of the construct, the content validity was assessed by the above mentioned panel of five experts, through examination, discussion and adaptation [31]. An initial assessment sheet with 20 items and five domains was proposed. Each item was ranked using a 3-point scale for relevance to the content domain, clarity, simplicity and whether or not the item should have been deleted. Both a content validity index (CVI) for each item [32] and a content validity ratio (CVR) for total scale according to Lawshe scores [33] were calculated.

#### 2.4.3. Internal Consistency

The internal consistency was evaluated by computing Cronbach’s alpha coefficients, using item responses from the 16 subtests obtained by the 505 participating children. Alphas of 0.7 or greater provided evidence of adequate internal consistency [34,35]. The average inter-item correlation was evaluated to estimate how much the items vary together on average. The item-test correlations were evaluated to show how highly correlated each item was with the overall scale; correlations below 0.2 are considered a cut-off point for discarding an item [36]. In order to estimate the value of these correlations with each item excluded, the item-rest correlations were also calculated.

#### 2.4.4. Construct Validity

In order to assess construct validity of the developed scale, a Structural Equation Modeling (SEM) analysis was conducted. This technique allows reducing the number of observed variables into a smaller number of latent variables by examining the covariation among the observed variables [37]. The authors used SEM also to estimate higher-order confirmatory factor analysis (CFA) models. The preschool-FLAT structure was then analyzed based on the 505 data collected and it was ascertained which model fitted the data better. The *sem* command was used in STATA to perform such analyses. Statistical significance was accepted at *p* < 0.05.

We tested four models. Model 1 (16-item uni-dimensional measurement model), in which the 16 items were assumed to be the indicator of a single latent factor that was FL. Model 2 (a higher-order model), in which the number of first-order latent factors was set to five, reflecting the five domains of the original structure, and the second-order latent factor was FL. Model 3 was similar to Model 2, but included four first-order latent factors, corresponding to the sum of first and second initial domains (leaving the other domains as they initially were set). The reason for this last model choice was that the four items excluded by the experts’ panel were in the first two domains, which at the end included two items each (instead of four). The fourth Model took into account a bi-dimensional measurement model where the 16 items could be determined by two latent variables, which were “knowledge” and “skills” (16-item bi-dimensional measurement model).

The goodness fit of the model was evaluated by using different indices, including: the comparative fit index (CFI), with a value ≥0.9 as a good fit; the root mean square error of approximation (RMSEA), which tests the fit of the model to the covariance matrix and has a value of 0.05–0.08 as an acceptable fit and <0.05 as a good fit; the standardized root mean squared residual (SRMR), which is the square root of the discrepancy between the sample covariance matrix and the model covariance matrix, with an acceptable fit value of 0.08 or less; and the relative χ^2^ (χ^2^/degrees of freedom) with a value of 3 or less considered as acceptable [38,39,40,41,42]. Moreover, Akaike information criteria (AIC) and Bayesian information criteria (BIC) were reported to compare the three models; the model with the smaller AIC is more likely to be replicated, has fewer parameter, and fits better [43].

The examination of the standardized coefficients of hypothesized relationships was performed together with the standard errors.

#### 2.4.5. Discriminant Validity

The discriminant validity was examined by comparing scores obtained by a convenient subgroup of 100 children out of the 505 who participated in the laboratory sessions (Test Group, “TG”), to scores of an additional group of children from one of the schools involved who did not attend any laboratory (*n* = 27) (Control Group, “CG”). This sample was recruited from a mixed socio-cultural environment.

FL scores, generated by summing up the scores of the single domains, and the scores of the single domains were compared in the TG and CG through unpaired Student’s t-test. Statistical significance was accepted at *p* < 0.05.

## 3. Results

### 3.1. Sample Characteristics

Sample Characteristics are showed in Table 1.

### 3.2. Content Validity

Questions agreed on by fewer than 3 experts were excluded. Experts came up to the decision of deleting 4 redundant items from the first two Domains, thus reducing the total item number to 16. The CVI of the overall tool was 0.94 and the CVR for total scale was 0.88, this indicating a satisfactory level of agreement among experts and a good content validity.

### 3.3. Internal Consistency

The items of the scale hang together pretty well, with the alpha equal to 0.77 (Table 2) and the average inter-item covariance equal to 0.17. Cronbach’s alpha coefficient ranged from 0.73 to 0.76. Item-test correlations were all above 0.20, ranging from 0.35 to 0.66, showing that each item was moderately correlated to the overall scale and thus no item was discarded (Table 2). This was corroborated by the values of inter-rest correlations, which decreased if the scale was computed only with the other 15 items (Table 2).

### 3.4. Construct Validity

The 16-item uni-dimensional model (Model 1) fitted the data poorly, as well as the 16-item bi-dimensional model (Model 4), as shown by the fit indices (Table 3).

**Figure 1:**
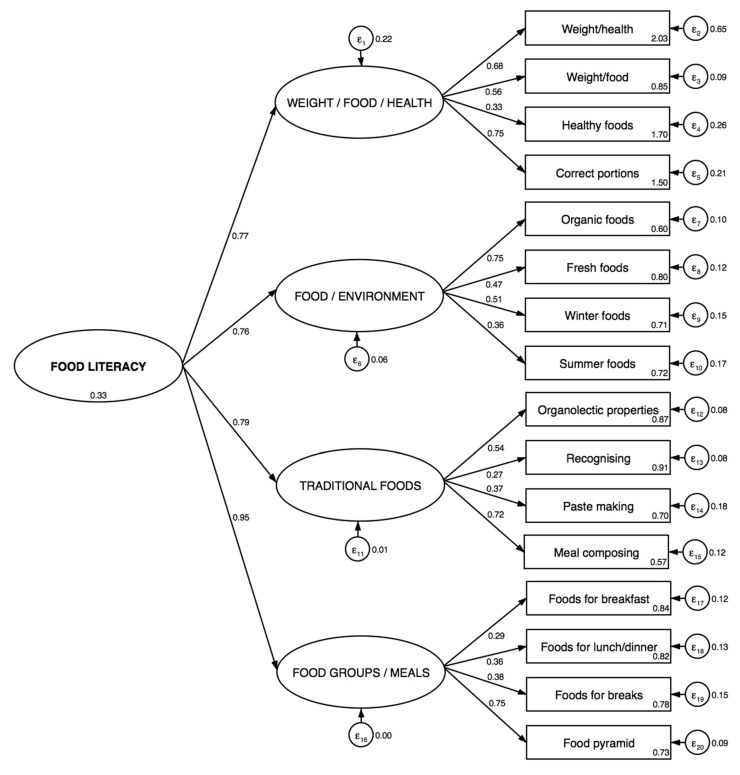
Four-factor structure of the preschool-FLAT scale obtained through a Structural Equation Modeling * (*n* = 505) (ε represents the error associated to the observed or latent variable).

The indices were improved with the 5-factor model (Model 2), but still with unsatisfactory validity of the scale (Table 3). Finally, the analysis showed that the identified 4-factor model (Model 3) fitted the data fairly, with a CFI = 0.939, RMSEA = 0.078, SRMR = 0.033, χ^2^/df = 2.97, AIC = 6778.0, and BIC = 6828.7 (Table 3). The four-factor structure of the scale is showed in Figure 1, together with standardized parameters associated to structural coefficients and standard errors; a very high coefficient (0.95) was found between FL and the dimension of food groups/meals, and moderately high coefficients were obtained between FL and the domains of traditional foods, weight/food/health and food/environment (0.79, 0.77, and 0.76, respectively).

### 3.5. Discriminant Validity

**Figure 2:**
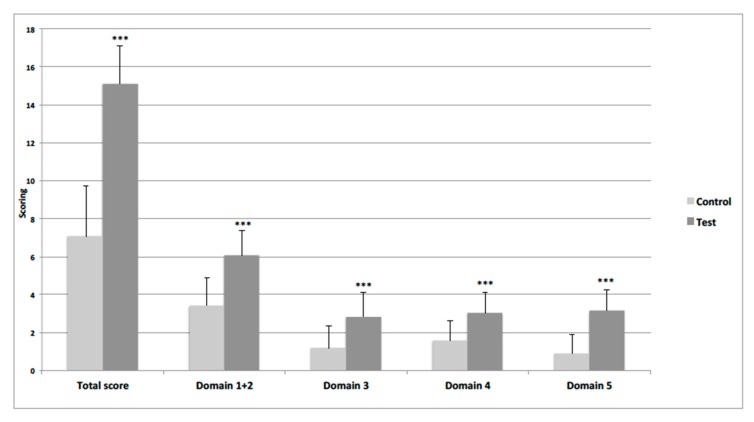
Comparison of preschool-FLAT scores derived from 4-factor model in control (*n* = 27) and intervention (n = 100). groups. Statistical significance was estimated through unpaired Student’s T test. *** *p* < 0.001.

Unpaired t-test revealed that there was a statistically significant difference between the FL score of TG and CG, with children who received intervention scoring higher than those who did not receive it (mean 15.1 vs. 7.1, *p* < 0.001) (Figure 2).

## 4. Discussion

The present study provides evidence for the internal consistency and validity of a new developed tool to assess FL in pre-schoolers. To the authors’ knowledge, no instrument in the literature has been developed so far to assess FL in such young children. Some authors developed FL questionnaires addressed to children in elementary schools and encouraged to perform these kinds of surveys in different age groups of children [22,23]; some others conceived an instrument for pre-schoolers, that anyway was aimed at assessing only knowledge of foods and their relative healthiness [44]. Thus, the development of a scale assessing pre-schoolers’ FL was required to guide the development and ensure effectiveness of nutrition related interventions [45].

FL has a complex nature including a social, cultural and political feature often difficult to measure, that should be considered as a structure with multiple dimensions [11,12,46]; for this reason a multi-dimensional tool should be used including a wide range of items. The preschool-FLAT was developed to specifically collect information on FL in pre-schoolers, and it is designed to reflect multi-dimensional aspects and to be appealing and relatively quick to assess through the use of pictures, images, and interview format instead of questionnaire. This assessment is made through a scale of 16 items, as agreed by the experts, which can describe four domains to obtain a satisfactory content validity. The good internal consistency was confirmed by the alpha, average inter-item correlation, item-test and item-rest correlations analyses (Table 2). There are reasons for believing that all of the 16 items are needed to measure different domains that underlay to a single concept, which is the FL, and catch the most important aspects of the construct (Figure 1).

Beyond this, an adequate construct validity of the instrument was demonstrated in this study (Table 3). The dimensions selected to construct the scale were representative both of knowledge and skills related to food and nutrition. However, when taking into consideration these two dimensions, the SEM demonstrated that they were poorly explained by those items. This is in contrast with what found in one scale developed for children from elementary schools which measured the cognitive domain (understanding and knowledge) and the skill domain (functional, food choice, interactive, and critical skills) with 6 subscales and confirmed the validity of this structure [23]. Instead, in our study, a 4-factor model was fairly consistent with our data, this being confirmed by acceptable values of absolute fit indices (relative χ^2^, RMSEA, and SRMR) and incremental and parsimony fit indices (CFI, AIC, and BIC); even though an initial 5-factor model of the FL scale was proposed, confirmatory analysis showed that four domains encompassing weight/food/health, food/environment, traditional foods, and food groups/meals were explained by the selected items (Figure 1).

Moreover, the group of children who participated in the intervention scored significantly better than control group, this demonstrating a good discriminant validity of the instrument. The intervention is thus supposed to be effective in increasing performances and cognitive abilities in preschoolers, and this could suggest to teachers that children in this age need to receive both education sessions and practical lab activities in the field of nutrition to take advantage in terms of learning skills.

Recent studies showed that FL can be interpreted as a specific form of health literacy [47], which was theorized by the Nutbeam’s model as being characterized by three levels as functional, interactive and critical [48]. The lowest functional level includes basic reading and writing skills necessary to understand and follow simple nutrition messages; the interactive level includes the skill of sharing nutritional information with others to promote healthy eating pattern; the critical level is the ability to analyze nutrition information critically, increase awareness, and participate in action to address barriers. It is intuitive that children in preschool age have not still developed the basic skills such as reading and writing, and at higher extent they cannot even be able to use health information to gain greater control over life events and situations. This implies that the authors of the present investigation have not applied this construct to the developed scale, in contrast with other studies addressed to scholars or adult/older people that were based on this hierarchical construct to measure validity of the scale for the assessment of FL [23,49,50]. The developed instrument, therefore, does not actually capture all aspects potentially relevant for the multifaceted concept of FL, but the authors believe that the chosen and analyzed items can cover all the possible knowledge and skills that can be reachable in the age range 3–6 years. Moreover, the preschool-FLAT represents a practical and appealing instrument able to catch different aspects of the food knowledge and skills in pre-schoolers; this tool is not limited to the simple recognizing of healthy and non-healthy food, but encompasses other aspects related to environment and tradition, which should always be taken into account in this kind of studies in order to create a food awareness since young age.

One limit of the proposed scale is that it was conceived for a population of young children in preschool age coming from the same geographical and urbanized area. It is clear that being FL highly influenced by culture and society, the role of sociocultural norms regarding health and eating have to be considered; thus, even though the developed scale was created and administered in a local Sicilian context, and then cannot serve as a universal tool, it can be easily adapted according to the different food traditions and social context [11,12]. Nevertheless, our sample was composed by children from low, medium and high socio-economic areas, this reflecting also the different cultural level of the participants, that were eligible to be included in the activities and in the assessment through the developed scale.

A limit of the study design is that the control sample, used for assessing the discriminant validity of the instrument, was quite lower (30%) than the test group, this probably affecting the power of the results. The small size of the control group depended mainly on the restricted budget availability and on the lacking perceived benefits of the eventual participation in the project from parents and children. However, the authors used them as control group because they showed socio-demographic characteristics similar to those of the intervention group.

Since there are not similar tools in the literature, it is quite difficult to compare the results of our analyses with others. Moreover, when the discriminant validity was estimated, we did not have a baseline score to which compare the scores obtained after the lab activities, this reducing the power of our results. Nonetheless, strength of this study is that SEM analysis was used, which is a confirmatory technique that can also be used for exploratory purposes. SEM, in comparison with CFA alone, extends the possibility of relationships among the latent variables and encompasses the components of a measurement model (essentially the CFA) and of a structural model [37].

## 5. Conclusions

Findings of the present study highlight that preschool-FLAT is a valid and consistent instrument, with good psychometric properties, and represents the only tool available in the literature aimed at assessing FL in pre-schoolers. Thus, it is a promising instrument that could be provided to stakeholders in order to monitor preschool children, identify possible determinants of low FL and implement preventive actions against food-related diseases in adult age. This instrument could be useful to schoolteachers with the aim of shaping pre-schoolers mind in the field of nutrition and diet, together with assessing their progresses and all criticisms, and consequent solutions to prepare them to deal with the primary school. Teachers could add this instrument in the school curriculum and dedicate only ten hours of their lessons during a whole school year, that is a very short and affordable time, so the teachers have not to subtract time to other programmed activities. The arguments treated during the lab sessions are also usual arguments treated by all teachers in one of the three years of the preschool curriculum, so there will not be any extra time for eventual teachers’ training. Moreover, budget request by the school is minor, since this tool consists only in five pages to be printed for each student; when computers are available within the school, all data can be reported into an excel database or inserted directly in excel. Therefore, the proposed tool can effectively support school operators help pre-schoolers in building their knowledge and skills at the same time since this early age, in order to develop a correct food literacy throughout their life course. This instrument is very easily adaptable to the preschool program as it perfectly fits within the school curriculum.

Moreover, the FLAT can support public health researcher in collecting important data to monitor and spread information on trends of pre-schoolers’ food literacy. Data collected through this tool could be widely used by the scientific community.

The next step for the future will be to assess the test-retest reliability of the instrument. After having confirmed validity and reliability, the authors wish that this tool could be adapted to other local realities within the country, in order to be widely used throughout the kindergartens of the territory. This spread collected information could be used to finally create a web platform where all the gathered data could be shared with universities and local government public health departments, which will be able to statistically elaborate them and provide information at regional and national level.

## Figures and Tables

**Table 1:** Characteristics of the preschool-FLAT (Food Literacy Assessment Tool) sample (*n* = 505).

	***n***	**%**
Gender		
Male	271	53.7
Female	234	46.3
Age (years)		
≤47 mo (3 years)	81	16.0
48–59 mo (4 years)	200	39.6
≥60 mo (5–6 years)	224	44.4
School SEE		
Low/medium	282	55.8
Medium/high	223	44.2
Weight status		
Underweight	53	10.5
Normalweight	336	66.5
Overweight	79	15.6
Obese	38	7.4
	**mean**	**SD**
Age (months)	57.3	10.1
Weight (Kg) ^1^	19.4	3.9
Height (m) ^2^	1.1	0.08
BMI (Kg/m^2^)	16.3	2.5
Scores		
Overall FL	14.9	3.8
Domain 1	3.0	1.3
Domain 2	3.2	1.0
Domain 3	2.9	1.2
Domain 4	3.0	1.2
Domain 5	3.0	1.2

SEE = socio-economic environment; BMI = body mass index; FL = food literacy. ^1^ Weight was collected by teachers through a SECA scale (precision 500 g and maximum capacity 150 Kg).^2^ Height was collected through a professional stadiometer (precision 0.1 cm).

**Table 2:** Cronbach’s α coefficients, item-test correlation and item-rest correlation for the preschool-FLAT scale (*n* = 505).

Item Number	Item Name	Item Description	Item Knowledge/Skill	Cronbach’s α	Item-Test Correlation	Item-Rest Correlation
Item 1	Weight/health	Weight status perception	Knowledge	0.7504	0.4981	0.3882
Item 2	Weight/food	Association of factors to weight status	Knowledge	0.7561	0.4381	0.3213
Item 3	Healthy foods	Healthy foods knowing, recognizing and naming	Knowledge	0.7608	0.3865	0.2650
Item 4	Correct portions	Correct portions knowledge	Knowledge	0.7470	0.5331	0.4274
Item 5	Organic foods	Be able to compose a meal with organic foods	Skill	0.7532	0.4686	0.3551
Item 6	Fresh foods	Be able to compose a meal with fresh foods	Skill	0.7539	0.4608	0.3465
Item 7	Winter foods	Be able to compose a meal with winter foods	Skill	0.7528	0.4729	0.3598
Item 8	Summer foods	Be able to compose a meal with summer foods	Skill	0.7545	0.4551	0.3401
Item 9	TF Organolectic properties	Recognizing organoleptic properties of foods and draw/paste those foods in the sheet	Skill	0.7474	0.5284	0.4220
Item 10	TF Recognizing	Guess the food from the description and draw/paste it in the sheet	Knowledge	0.7598	0.3978	0.2773
Item 11	TF past making	Be able to make a dough with flour and water and draw/paste	Skill	0.7623	0.3696	0.2469
Item 12	TF meal composing	Be able to assemble a meal with typical/regional foods	Skill	0.7431	0.5719	0.4717
Item 13	Foods for breakfast	Recognize food suitable for breakfast	Knowledge	0.7637	0.3541	0.2300
Item 14	Foods for lunch/dinner	Recognize food suitable for lunch/dinner	Knowledge	0.7501	0.5008	0.3910
Item 15	Foods for breaks	Recognize food suitable for breaks	Knowledge	0.7576	0.4221	0.3038
Item 16	Food pyramid	Be able to put the food in the right level of the food pyramid	Knowledge	0.7338	0.6615	0.5760
Overall				0.7649		

TF = Traditional Foods.

**Table 3:** Results of the Structural Equation Modeling of the Preschool-FLAT (*n* = 505).

	Model 1 Latent Variable: Food Literacy; Observed Variables: 16 Items	Model 2 First-Order Latent Variables: Five Domains; Second-Order Latent Variable: Food Literacy; Observed Variables: 16 Items	Model 3 First-Order Latent Variables: Four Domains; Second-Order Latent Variable: Food Literacy; Observed Variables: 16 Items	Model 4 First-Order Latent Variables: Knowledge, Skills; Second-Order Latent Variable: Food Literacy; Observed Variables: 16 Items
CFI	0.521	0.747	0.939	0.556
RMSEA	0.123	0.094	0.078	0.119
SRMR	0.095	0.078	0.033	0.092
χ^2^/df	8.65	5.47	2.97	8.15
AIC	9871.065	9506.218	6778.033	9813.594
BIC	10,073.654	9751.012	6828.727	10,020.403

CFI = Confirmatory factor index; RMSEA = root mean Square error of approximation; SRMR = standardized root mean squared residual; χ^2^ = chi-square; df = degrees of freedom; AIC = Akaike information criteria; BIC = Bayesian information criteria.

## Supplementary Materials

The following are available online at https://www.mdpi.com/1660-4601/17/8/2759/s1, Supplementary S1: Preschool-FLAT sheets translated in English with description of the five domains’ objectives, activities and evaluation system.
