# Supplementary material for: Validity and Internal Consistency of the Preschool-FLAT, a New Tool for the Assessment of Food Literacy in Young Children from the Training-To-Health Project
Source: Int J Environ Res Public Health. 2020 Apr 16;17(8):2759. doi: 10.3390/ijerph17082759 (PMC7215692; doi:10.3390/ijerph17082759)
Supplement: Supplementary file 1 [file ijerph-17-02759-s001.pdf]

SURNAME AND NAME	SCHOOL	GRADE

FOOD LABORATORY
MODULE 1 – BODY WEIGHT AND FOODS

**General objective**	Understand the meaning of overweight/obesity and their main determinants
**Specific objectives**	- Know the meaning of overweight/obesity<br>- Discriminate between figures of different body weight<br>- Identify the main determinants of overweight and obesity
**Short introduction**	Body weight and its classification. The relationship between food intake and obesity.
**Activity 1**	Prepare some profiles of children with different weight (under, normal, over weight/obese) and ask children to observe them.<br>Ask them which profile they think is associated to health, and ask them to tick the correct smile close to each profile.
**Activity 2**	Prepare a sheet with one profile of obese child and an empty space beside.<br>Ask pupils to draw and paint on a blank sheet of paper a food that they link to the obese child, cut out and paste in the empty space beside the correct profile.

EVALUATION

Tick the smile corresponding correctly to the profile.<br>Score = 1 point per each profile correctly perceived.		
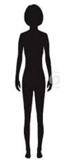	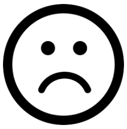 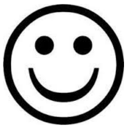	
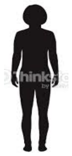	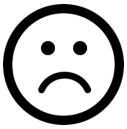 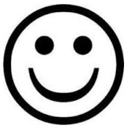	
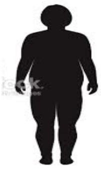	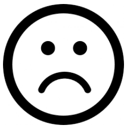 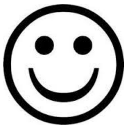	
	**SCORE**	

Paste the drawn and painted food that you link to the obese profile.<br>Score = 1 point if the drawing is correct in relation to the obese profile.		
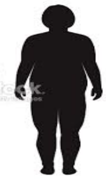		
	**SCORE**	

**TOTAL SCORE**	

SURNAME AND NAME	SCHOOL	GRADE

FOOD LABORATORY
MODULE 2 – FOOD QUALITY/QUANTITY AND HEALTH

**General objective**	Understand the relationship between the food quality/quantity and health.
**Specific objectives**	- Recognise and name the healthy and the non-health foods.<br>- Experience the weighing of some foods, and discriminate between the different quantities.
**Short introduction**	The main classes of foods. Healthy and non-healthy foods. Correct quality and quantity of the foods in the diet.
**Activity 1**	Prepare a sheet with different healthy and less or non-healthy foods.<br>Ask pupils to choose two foods, one healthy and one non-healthy, and draw, paint and cut out them on a blank sheet.<br>Ask pupils to paste the two chosen foods on the empty space of the sheet beside the correct smile (healthy=happy smile; non-healthy=sad smile).
**Activity**	Take some foods (fruit, pasta, etc.) and make some example of small, medium and big portion sizes.<br>Ask children to weigh the foods, and ask them if the portions are small, medium or big.<br>Ask children to draw, paint and cut out two portions of food on a blank sheet, one medium and one big.<br>Ask pupils to paste the two portions on the empty space of the sheet beside the correct smile (medium portion=happy smile; big portion=sad smile).
**Activity 3**	Ask children to associate to some foods the colour of health (yellow, orange, red from the fruit and vegetables) and to other the colour white (sugar, butter, etc.)<br>Ask them to draw and paint these foods.

EVALUATION

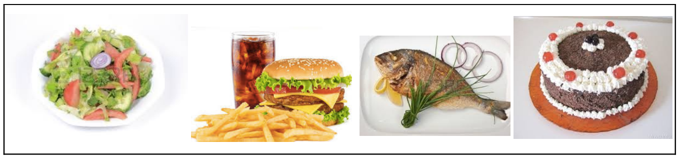

Paste two foods, one healthY and one non healthy in the empty space beside the correct smile.
Score = 1 point per each food pasted in the correct space.

	**SCORE**	

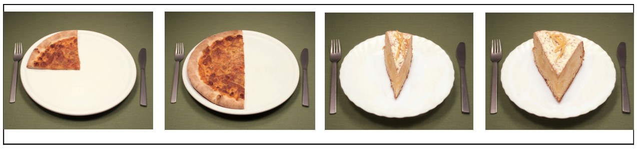

Paste two portions, one correct and one non correct corresponding to the smile.
Score = 1 point per each portion pasted in the correct space.

	**SCORE**	

**TOTAL SCORE**	

SURNAME AND NAME	SCHOOL	GRADE

FOOD LABORATORY
MODULE 3 – EAT ORGANIC AND FOLLOW SEASONS!

**General objective**	Understand the relationship between food and environment (organic food, seasonality)
**Specific objectives**	Know the meaning of organic food; recognise packed organic foods. Identify foods from each season.
**Short introduction**	Organic foods: definition; advantages for the environment and health; European logo for packed foods. Seasons and their foods.
**Activity 1**	Show to the children some packed organic products and non-organic products.<br>Prepare some sheets with the images of these foods. Ask them to cut them out and paste on a sheet gathering them to compose a meal.<br>If they will have composed a meal by choosing all the organic foods, they will have given the correct answer.
**Activity 2**	Show to the children fruit and vegetables typical from each season.<br>Ask them to compose a meal by adding foods from the winter season.

EVALUATION

Compose a meal by choosing foods among those shown organic and non-organic.
Score = 1 point if they choose all organic foods.

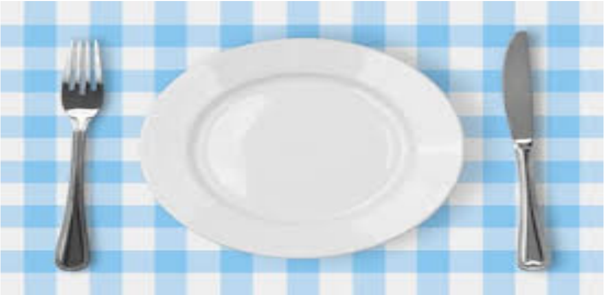

SCORE

Compose a meal by choosing foods among those shown fresh from the garden and packed.
Score = 1 point if they choose foods fresh from the garden.

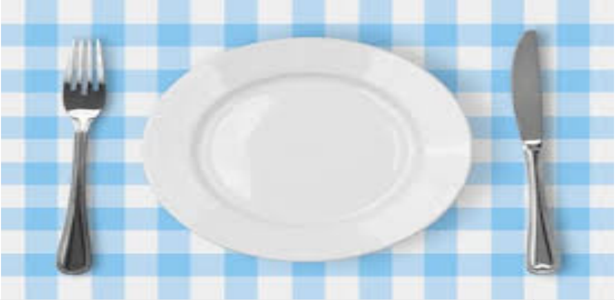

SCORE

Compose a “winter meal” by choosing foods among those shown.
Score = 1 point if they choose winter foods.

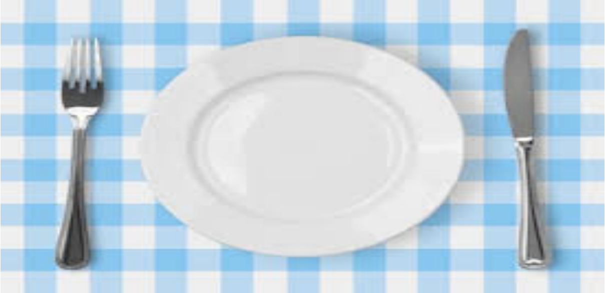

SCORE

Compose a “summer meal” by choosing foods among those shown.
Score = 1 point if they choose summer foods.

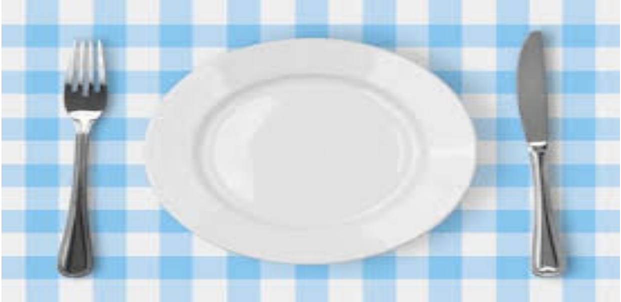

SCORE

TOTAL SCORE

SURNAME AND NAME	SCHOOL	GRADE

FOOD LABORATORY
MODULE 4 – LET’S KNOW TRADITIONAL SICILIAN FOODS

**General objective**	Promote knowledge and consumption of quality Sicilian agricultural foods, that are connected to the tradition and culture of the territory and that are obtained by respecting the environment.
**Specific objectives**	Know the Mediterranean Diet. Learn that Sicily is included in the Mediterranean Diet countries and has several typical genuine products.
**Short introduction**	Brief notes on the geography of the Mediterranean countries, and Sicily in particular. Typical foods of the Mediterranean Diet and the most common Sicilian agricultural products.
**Activity 1**	Show to the children, and in some cases let them smell and touch, some of the agricultural Sicilian products (extra virgin olive oil, oranges, herbs, olives, almonds, cheeses, etc.).<br>Ask them to comment on the different smells, tastes, shapes, colours.<br>Ask them to indicate on a sheet the smile corresponding to the perception during the sensorial experience.<br>Blindfold the children, and let them smell and touch one food previously shown, asking them to identify the correct one. Mark on the sheet if they guessed it.
**Activity 2**	Divide children in two teams. The operator should describe different characteristics of a typical Sicilian food and of the Mediterranean tradition, and the teams have to guess what food it is.
**Activity 3**	Make the children knead water and flour. Ask them to create shapes that represent Sicilian foods.
**Activity 4**	Prepare a sheet with different typical Sicilian and non-typical foods. Ask them to compose a meal by choosing the typical foods.

EVALUATION

Ask the children to draw (or paste) the food they recognised while they were blindfolded.
Score = 1 point if they recognise it.

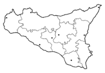

SCORE

Ask the children to draw (or paste) the food that was described by the teacher.
Score = 1 point if they guess it.

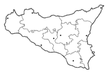

SCORE

Ask the children to draw (or paste) the food created with the dough of water and flour.
Score = 1 point if they reproduce them correctly.

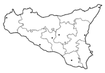

SCORE

Compose a typical Sicilian meal by choosing foods among those shown.
Score = 1 point if they choose the correct foods.

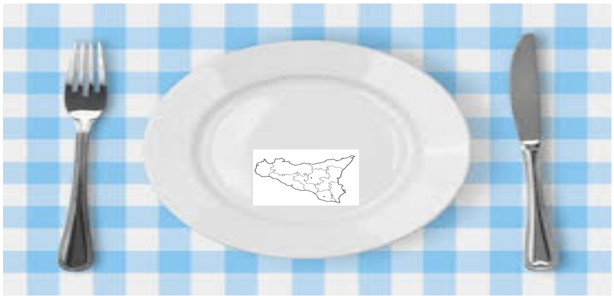

SCORE

TOTAL SCORE

SURNAME AND NAME	SCHOOL	GRADE

FOOD LABORATORY
MODULE 5 – LET'S BUILD THE FOOD PYRAMID

**General objective**	Favour building healthy food habits.
**Specific objectives**	Know the different food groups and their suggested distribution in a day.<br>Know the food pyramid.
**Short introduction**	Food classification. The daily meals. The food pyramid.
**Activity 1**	Prepare a sheet with the foods suitable for breakfast, morning/afternoon break, and lunch/dinner.<br>Ask the children to paint the foods suitable for each of the described meal.
**Activity 2**	Prepare a blank poster and cut with a shape of triangle. Draw different lines for the levels of the pyramid.<br>Ask children to draw, paint and cut out a different food.<br>Create a path that leads each child to paste the food in the correct pyramid level, by respecting the pyramid rules.

EVALUATION

Paint the foods suitable for breakfast.
Score = 1 point if foods are painted correctly.

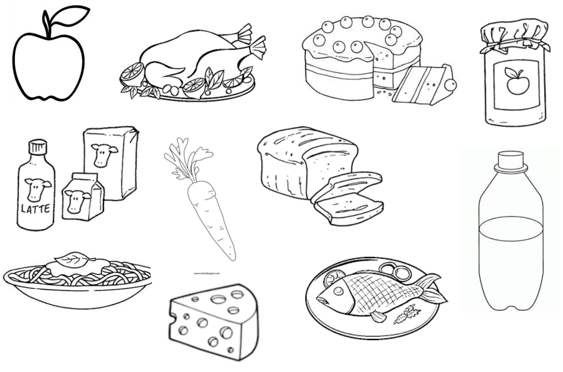

SCORE

Paint the foods suitable for lunch/dinner.
Score = 1 point if foods are painted correctly.

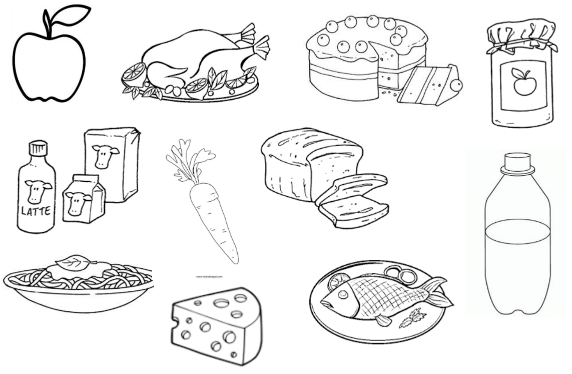

SCORE

Paint the foods suitable for the morning or afternoon break.
Score = 1 point if foods are painted correctly.

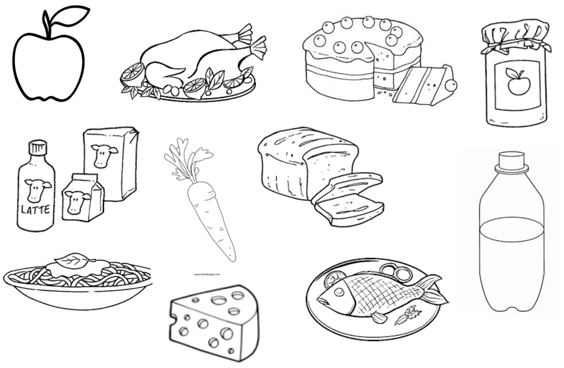

SCORE

Paste the food in the correct level, by respecting the pyramid rules.
Score = 1 point if the food is pasted in the correct level.

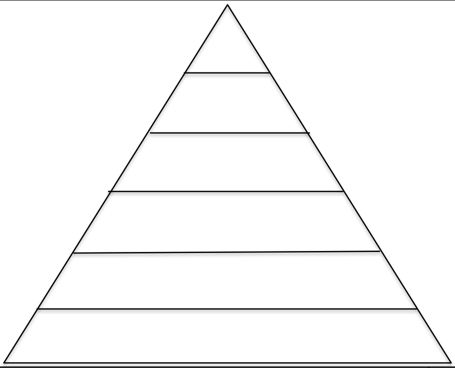

SCORE

**TOTAL SCORE**	
